# Histological assessment of the efficiency of rabbit serum in healing skin wounds

**DOI:** 10.14202/vetworld.2019.1650-1656

**Published:** 2019-10-28

**Authors:** Abeer Ahmed Majeed, Dhyaa Ab. Abood

**Affiliations:** 1 Department of Surgery and Obstetrics, College of Veterinary Medicine, University of Baghdad, Baghdad, Iraq; 2 Department of Anatomy and Histology, College of Veterinary Medicine, University of Baghdad, Baghdad, Iraq

**Keywords:** healing, rabbits, serum, skin, wounds

## Abstract

**Aim:**

This study aimed to investigate the impact of rabbit serum on skin wound healing with the help of histological examination.

**Materials and Methods:**

A total of ten indigenous rabbits were used in this study. The animals were divided into two groups: control and serum- treated. The histological assessment was done with a paraffin embedding technique and the histological sections were stained with H&E stain.

**Results:**

Severe infiltration of polymorphonuclear leukocytes with severe fibrin deposits were seen in serum treated group at 2 days post-injury; at 7 days post-injury the changes revealed moderate fibroplasia, fibrin deposit and severe infiltration of both mononuclear and polymorphonuclear leukocytes; at 14 days post-injury, there were marked epithelization and dermal deposition of collagen fibers; and at 21 days post-injury, the epidermis completed epithelization and the dermis showed neither fibroplasia nor infiltration of mononuclear and polymorphonuclear leukocytes.

**Conclusion:**

The results indicated that rabbit’s serum can prevent wound infection, accelerate epithelialization and cutaneous regeneration with less granulation.

## Introduction

The cutaneous tissue which covers the entire body is composed of keratinized stratified squamous epithelium that represents the epidermis and the dermis, which is composed of interconnected collagen bundles and elastic fibers [1,2]. The skin wound is a discontinuity of the soft tissues including cutaneous tissue and the infected wound needs a longer time to repair their associated complications. Wound healing is a complex process that is composed of reactions and interactions between cells and biochemical mediators that attempt to repair the injured area and begin immediately following an injury [3]. Skin wounds usually occur by accidental trauma (burns, lacerations, or abrasions), which lead to complications such as delay healing and infection [4]. Serum is a biological body fluid and contains several factors such as growth hormone and insulin-like growth factor [5,6]. Serum is widely used in culturing media of different origins and it is essential for cell growth, metabolism, and enhanced proliferation because it has most factors required for cell attachment, growth, and proliferation [7]. The serum has previously been reported to exhibit potent antimicrobial, antioxidant, and anti-inflammatory activities. During wound healing, these biological properties play a crucial role in supporting the formation of new tissue around the injured skin in the recovery process [8].

Wound repair involves several events that include some complicated interactive processes between the cells and their mediators which are essential for wound healing [3,9]. Using biologically safe and natural compounds are effective, especially when they have low cost and better toleration by patients [9]. Several surgical techniques have been used in skin wound healing including dressings, and most of these techniques depending on the bioproducts derived from animal sources (xenografts) (for example, dermal tissue, intestinal submucosa, pericardial, and amniotic membranes) [10-13]. The serum contains a group of proteins that do not involve blood clotting electrolytes, hormones, and antibodies that have antimicrobial and anti-inflammatory activities [8]. Many studies have investigated the role of serum in wound healing, and for many decades, the bovine serum has been used as an essential component for the growth of animal cells in culturing sold media and liquid media [14]. Moreover, autologous has been successfully used in the healing of 43.8% of the persistent corneal epithelial defects within 2 weeks [15]. The activity of subcutaneous injected dialyzed serum has revealed marked angiogenic effects and it has been reported as an effective factor in stimulating vascular permeability in wound repair; furthermore, the vascular permeability allows different cytokines and growth factors to reach the wounded area on the vascular permeability for stimulating angiogenic activity [16]. Serum amino-terminal properties type III procollagen (PIIINP) is important for tissue repair and post-operative increase in serum PIIINP levels is partly the result of tissue repair and partly the result of whole-body turnover of type III collagen [17].

This study aimed to investigate the impact of the rabbit serum on skin wound healing with the help of histological examination.

## Materials and Methods

### Ethical approval

The design of the present study was approved by the Animal Care and Use Committee at the College of Veterinary Medicine, University of Baghdad. Baghdad, Iraq.

### Experimental animals and wound induction

A total of 10 healthy indigenous rabbits (age: 8-10 months old and weight 1.5-2 kg) were treated by intramuscular injection of 20,000 IU/kg-10 mg/kg body weight penicillin-streptomycin 30 min before making the wound. Rabbits were treated by intramuscular injection of 0.05 mg/kg acepromazine and the local anesthesia was accomplished with ketamine HCl (35 mg/kg) and xylazine (5 mg/kg). Three square full-thickness skin wounds (3 cm×3 cm) have created on the dorsal thoracic sides [11]. The animals were divided into two main groups: Control and serum-treated groups (STGs). All wounds were remained as opened wounds. The control wounds were treated with a single injection of 2 ml normal saline, while the wounds of the second group were treated by intradermal injection of 2 ml serum at the site of incision. In both groups, the wound sites were covered by sterile gauze. The bandages were changed gently after 48 h [11]. All animals were observed daily for any complications.

### Preparation of serum

Blood samples were collected from the ear vein in test tubes without anticoagulants. The blood samples were completely set or clotted by storing at 4°C overnight. Serum was obtained by centrifugation at 3000 rpm for 5 min and kept at 20°C until required.

### Histopathological evaluations

Samples of skin (1 cm^2^) were obtained from the sites of injuries at 2, 7, 14, 21, and 28 days after wounding. The samples were washed up with normal slain twice and fixed in 10% neutral buffer formalin for 72 h. The tissue specimens were processed with the paraffin technique, sectioned at 5-7 μm, and stained with hematoxylin and eosin [18]. The histopathological assessment graded the changes as mild, moderate, and severe. Inflammatory reactions included inflammatory exudate and infiltration of mononuclear or polymorphonuclear leukocytes and epidermal or dermal remodeling phase included reepithelization, fibroplasia, and collagen depositions [11].

### Statistical analysis

Data were expressed as mean ± standard error. Statistical analysis was carried out on the load-bearing data using two-way analysis of variance and the least significant difference. p<0.05 was considered as statistical significance differences.

## Results and Discussion

### On day 2

**Figure-1:**
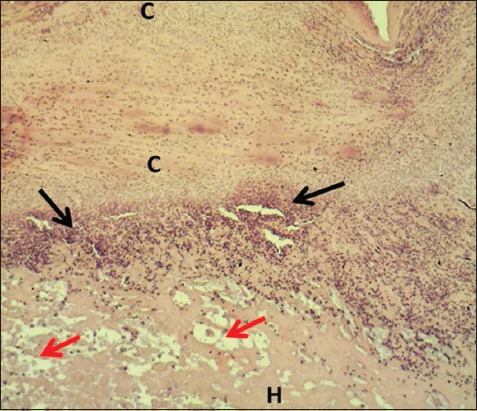
Section of skin (2 days post-injury in control group) shows clot (C), inflammatory zone (black arrows), edema (red arrows), and hemorrhage (H). Hematoxylin and eosin stain, 40×.

**Figure-2:**
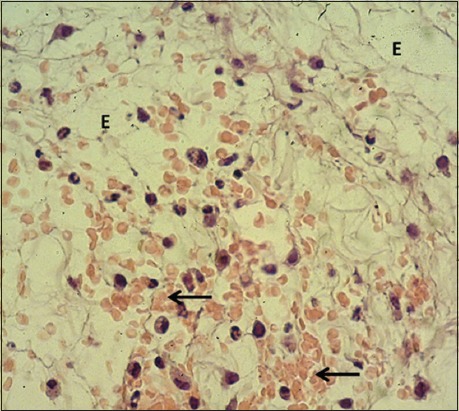
Magnified section of skin (2 days post-injury in control group) shows edema (E) and hemorrhage (arrows). Hematoxylin and eosin stain, 400×.

**Figure-3:**
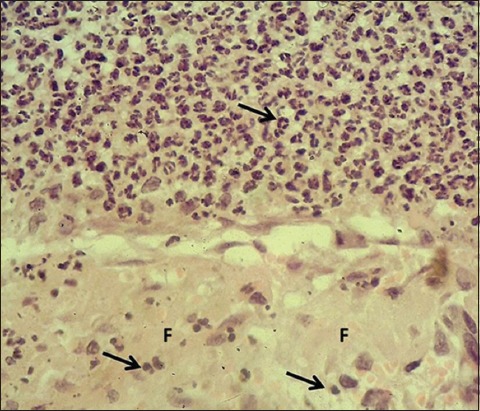
Section of skin (2 days post-injury in serum-treated group) shows mononuclear and polymorphonuclear leukocytes (arrows) and severe fibrin deposit (F). Hematoxylin and eosin stain, 400×.

**Figure-4:**
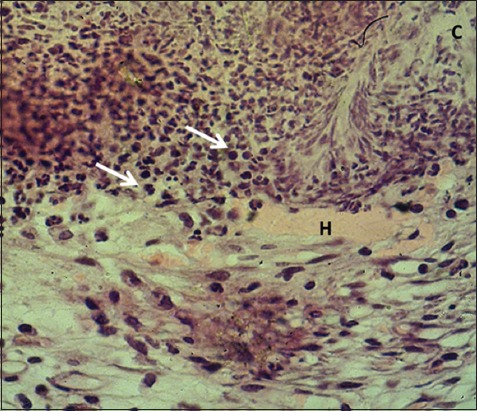
Magnified section of skin (2 days post-injury in serum-treated group) shows clot (C), hemorrhage (H) with polymorphonuclear and mononuclear leukocytes (arrows). Hematoxylin and eosin stain, 400×.

In the control group, the skin sections were covered with a very thick layer of the clot separated from underneath dermis by a zone of inflammatory cells composed of polymorphonuclear leukocytes; the dermis showed multiple hemorrhagic foci and edema. The sections of STG showed the same changed along with marked dermal fibrin deposit with severe infiltration of mononuclear leukocytes and polymorphic nuclear leukocytes (Figures-1-4). The results revealed a thick layer of the clot which covered the injured skins of both control and serum-treated groups. This result was associated with the role of platelet aggregate and seals the bleeding through forming thrombi at the wounds [19,20]. In addition, our results revealed the infiltration of polymorphonuclear leukocytes forming a zone of inflammatory cells; this is in line with the findings of Al-Falahi *et al*. [11], Mohammed [21], Kotani and Sakane [22] that the role of neutrophils in releasing chemokines and cytokines which induce oxidative stress. Our results revealed no sign of fibrogenesis in the early stages of wound healing. This observation is similar to the case of amniotic membrane dressing technique recorded by Al-Falahi *et al*. [11], Mohammed *et al*. [21], Albannaa *et al*. [23], and Duarte and Araujo [24]. Moreover, no sign of inflammatory reactions (hemorrhage and edema) was seen in serum-treated group, which suggests the presence of activities of serum antibacterial agents and immunoglobulin [8,25,26]. The fibrin deposit was the marked inflammatory exudate in serum-treated group, which is similar to the results recorded by Al-Falahi *et al*. [11] and Mohammed *et al*. [21] through using amniotic membrane technique. This suggests that the fibrin deposit can help in limiting the spread of tissue infection to the surrounding intact tissue. A similar observation was witnessed by Lima *et al*. [27] who investigated the role of ascorbic acid (Vitamin C) in healing skin wounds in rats and reported less inflammatory reaction, fewer macrophages, and well angiogenesis.

### On days 7 and14

**Figure-5:**
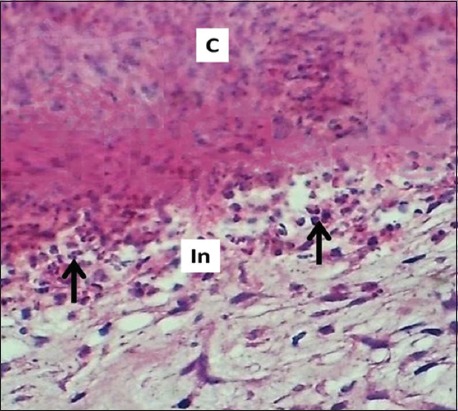
Section of skin (7 days post-injury in control group) shows clot (C), inflammatory zone (In), mononuclear and polymorphonuclear leukocytes (arrows). Hematoxylin and eosin stain, 400×.

**Figure-6:**
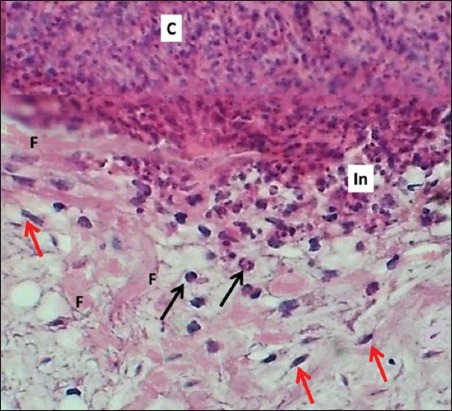
Section of skin (7 days post-injury in serum-treated group) shows clot (C), mononuclear and polymorphonuclear leukocytes (black arrows), inflammatory zone (In), fibrin deposited (F), and fibrogenesis (red arrows). Hematoxylin and eosin stain, 400×.

**Figure-7:**
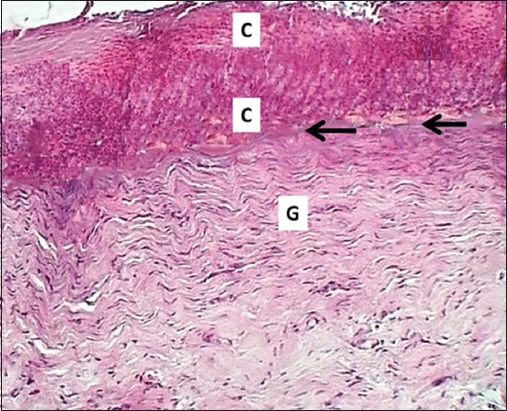
Section of skin (14 days post-injury in control group) shows clot (C), inflammatory zone (black arrows), and immature collagen fibers (G). Hematoxylin and eosin stain, 100×.

**Figure-8:**
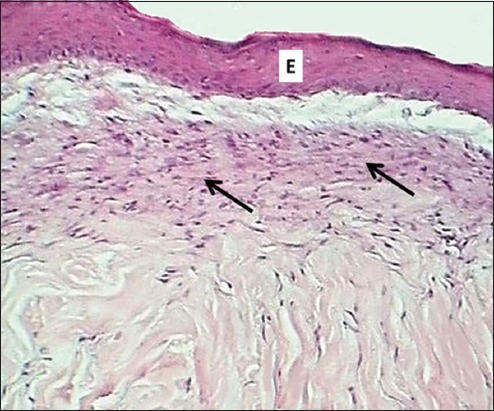
Section of skin (14 days post-injury in serum-treated group) shows epithelization (E) and deposit of immature collagen (arrows). Hematoxylin and eosin stain, 100×.

At day 7 post-injury, the sections of the control group revealed a thick layer of clot followed by a zone of inflammatory cells that composed of polymorphonuclear and mononuclear leukocytes. In serum-treated group, the sections revealed marked fibrin deposition between the zone of inflammatory cells and the dermis. The dermis revealed marked fibroplasia which composed of fibroblast and angiogenesis (Figures-5 and 6). These results indicated that the serum a mixture contains protein (fibrin) for clotting and some bioactive molecules which play an important role in wound healing. This finding is similar in role of platelet growth factors and cytokines [28,29]. On day 14 after serum injection, the sections of serum-treated group showed marked reepithelization of the epidermis which revealed epithelial height about 23.9±0.2, while the dermis showed marked deposition of immature collagen fibers (Figures-7 and 8). The present results revealed marked reepithelization in serum-treated group which agrees with the results recorded by Mohammed *et al*. [21], on day 7 after injury in rabbits dressed with the canine amniotic membrane. These observations suggest that different animal species give various effective stages of healing. These results disagree with those of Duarte and Araujo [24] in rabbit’s wound dressings with amniotic membrane. However, Albannaa *et al*. [23] recorded marked reepithelization on day 3 in wounded rabbits dress with amniotic membrane, this individual variation suggests that this could be related to the site of application (external wound or internal). On the other hand, the results of serum-treated group on day 14 after injury revealed the presence of densely packed bundles of collagen fibers and less mature granulation tissue in wound repair that suggests increased tensile strength in the repaired wound. The less granulation tissue is related to fibrosis reducing activities that take place by certain serum factors that related with mechanism of evaluated gene expression at which the procollagen (alpha-1) was inhibited and activated myofibroblasts, leading to a substantial collagen production [30-32]. On the other hand, Sarpooshi *et al*. [33] used topical Vitamin C in the treatment of burn wounds and reported that Vitamin C creates fresh and abundant mature granulation tissues.

### On day 12

**Figure-9:**
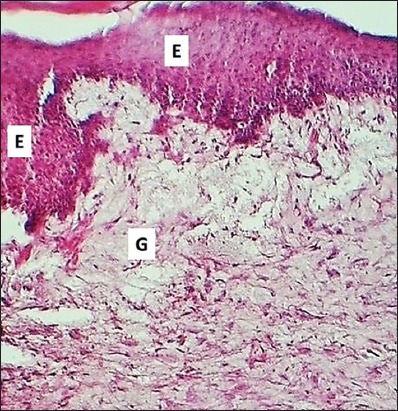
Section of skin (21 days post-injury in control group) shows thick epithelium (E) and immature granulation tissue (G). Hematoxylin and eosin stain, 100×.

In the control group, the skin sections showed severe epithelization of epidermis forming a layer of the stratified squamous epithelium that revealed epithelial height about 68.2±1.8 µm; the underneath dermis showed severe fibroplasia of fibroblasts and fibrocytes with immature collagen bundles, with no infiltration of polymorphonuclear and mononuclear leukocytes; and the sections showed that there were no sebaceous glands and hair follicles formation (Table-1). In serum-treated group, the skin sections revealed complete epithelization of the epidermis, which composed of mature stratified squamous epithelium with significant epithelial height (88.3±0.8 µm), compared to that of the control group (Table-2); in addition, the dermis composed of mature irregular dense collagenous connective tissue that contains newly formed hair follicle (Figures-9 and10). This result suggests the differentiation of epithelial cells of the epidermis for the presence of active biosubstances which promote such act as moistures maintenance, promoting epithelialization, and wound contraction. This is similar to the report by Jangpromma *et al*. [8] that highlighted the ability of the serum to decrease the wound area and accelerate epithelialization throughout cells migration of the proliferative epithelial cells. The result of serum-treated group showed no scar formation which associated serum biosubstances that play an important role in regeneration of new tissue around the injured skin [8,34]. In comparison with other techniques such as pericardial membrane dressing technique [11] which indicates for marked proliferation of fibroblasts that play an important role in closure of wound, but this process is accompanied by hyper granulation tissue mentioned by Zahm *et al*. [35].

**Table 1:** Scoring of histological changes during healing in both control and STG.

Days	Fibrin deposited		Reepithelization		Fibroplasia		Mononuclear cells		Polymorphonuclear cells	
				
	Control	STG	Control	STG	Control	STG	Control	STG	Control	STG
2	None	Severe	None	None	None	None	None	None	Severe	Severe
7	None	Moderate	None	None	Mild	Moderate	Severe	Severe	Severe	Severe
14	None	None	None	Mild	Severe	Mild	Severe	Mild	Severe	Mild
21	None	None	Severe	Complete	Severe	None	None	None	None	None
28	None	None	Complete	None	None	None	None	None	None	None

STG=Serum-treated group

**Table 2:** Epithelial heights in control and serum-treated groups during the periods of experiment.

Period/days	Control/μm mean±SE	Serum treated/μm mean±SE
2	None	None
7	None	None
14	None	23.9±0.2
21	68.2±1.8	*88.3±0.8
28	99.1±0.9	*119.3±1.1

* Significant differences (p<0.05). SE=Standard error

**Figure-10:**
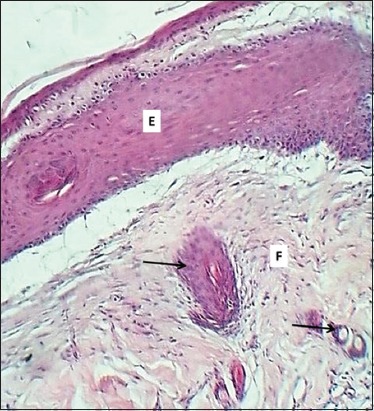
Section of skin (21 days post-injury in serum-treated group) shows epithelium (E), fibrogenesis (F), and newly formed hair follicles (arrows). Hematoxylin and eosin stain, 100×.

### On day 28

**Figure-11:**
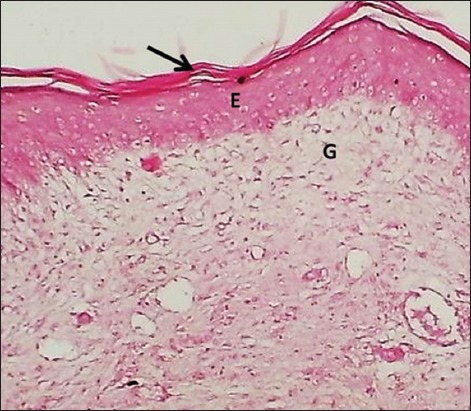
Section of skin (28 days post-injury in control group) shows keratin layer (arrow), epithelization (E), and granulation tissue (G). Hematoxylin and eosin stain, 100×.

**Figure-12:**
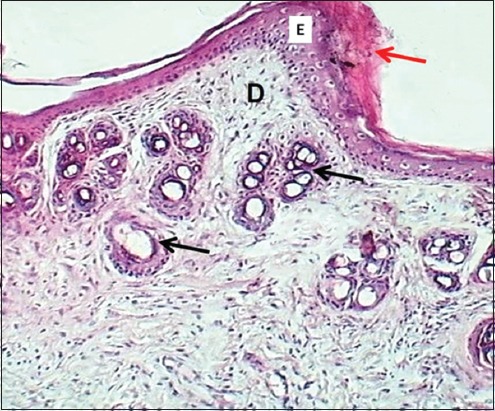
Section of skin (28 days post-injury in serum-treated group) shows keratin layer (red arrow), epithelium (E), dermis (D), and hair follicle (black arrows). Hematoxylin and eosin stain, 100×.

In the control group, the epidermis completed epithelialization with epithelial height (99.1±0.9 µm) and the dermis revealed marked granulation tissue which composed of few fibrocytes and no infiltration of leukocytes. In the serum-treated group, the skin sections showed normal cytoarchitecture of skin which indicates epithelial keratinization with significant epithelial height (119.3±1.1 µm); and the dermis showed the maturation of collagen bundles with the formation of sebaceous glands (Figures-11 and 12). The changes witnessed in this period were similar to those recorded by Al-Falahi *et al*. [11] and the absence of leukocytes with no fibroplasia agrees with the results of Duarte and Araujo [24] who used amniotic membrane dressing technique in infected wounds in rabbits.

## Conclusion

The results of this study indicated that the rabbit serum can prevent wound infection and accelerate the reepithelialization necessary for the formation of new skin. In addition, the healing process had less granulation tissue with marked formation of glands and hair follicles.

## Authors’ Contributions

AAM and DAA designed and carried out the study. Both authors read and approved the final manuscript.
